# *Lactobacillus fermentum* promotes adipose tissue oxidative phosphorylation to protect against diet-induced obesity

**DOI:** 10.1038/s12276-020-00502-w

**Published:** 2020-09-11

**Authors:** Youngmin Yoon, Gihyeon Kim, Myung-giun Noh, Jeong-hyeon Park, Mongjoo Jang, Sungsoon Fang, Hansoo Park

**Affiliations:** 1 Department of Biomedical Science and Engineering, Gwangju Institute of Science and Technology (GIST), Gwangju, Korea; 2 Genome and Company, Pangyo-ro 255, Bundang-gu, Seoungnam Korea; 3 Severance Biomedical Science Institute, BK21 PLUS Project for Medical Science, Gangnam Severance Hospital, Yonsei University College of Medicine, Seoul, Korea

**Keywords:** Obesity, Gene expression, Metabolic syndrome

## Abstract

The gut microbiota has pivotal roles in metabolic homeostasis and modulation of the intestinal environment. Notably, the administration of *Lactobacillus* spp. ameliorates diet-induced obesity in humans and mice. However, the mechanisms through which *Lactobacillus* spp. control host metabolic homeostasis remain unclear. Accordingly, in this study, we evaluated the physiological roles of *Lactobacillus fermentum* in controlling metabolic homeostasis in diet-induced obesity. Our results demonstrated that *L. fermentum-*potentiated oxidative phosphorylation in adipose tissue, resulting in increased energy expenditure to protect against diet-induced obesity. Indeed, oral administration of *L. fermentum* LM1016 markedly ameliorated glucose clearance and fatty liver in high-fat diet-fed mice. Moreover, administration of *L. fermentum* LM1016 markedly decreased inflammation and increased oxidative phosphorylation in gonadal white adipose tissue, as demonstrated by transcriptome analysis. Finally, metabolome analysis showed that metabolites derived from *L. fermentum* LM1016-attenuated adipocyte differentiation and inflammation in 3T3-L1 preadipocytes. These pronounced metabolic improvements suggested that the application of *L. fermentum* LM1016 could have clinical applications for the treatment of metabolic syndromes, such as diet-induced obesity.

## Obesity: Microbial defenders of metabolic health

The presence of particular bacterial species in the healthy gut microbiome can reduce the risk of obesity by modulating metabolic activity in host tissues. Several studies have suggested that intestinal microbes from the genus *Lactobacillus* can counteract weight gain and fat accumulation in humans and mice. Researchers in South Korea led by Sungsoon Fang at Yonsei University College of Medicine, Seoul, and Hansoo Park at the Gwangju Institute of Science and Technology have now examined the physiological effects of one such species, *Lactobacillus fermentum* LM1016, in mice. The researchers showed that this bacterium could reduce the risk of diet-induced obesity and fat accumulation in the liver, and identified relevant shifts in the activity of key metabolic and inflammatory pathways. If these findings are confirmed in humans, this bacterial strain could serve as an effective probiotic.

## Introduction

Obesity is defined as abnormal or excessive fat accumulation. Approximately 30% of the world’s population is considered obese or overweight, and the global prevalence of obesity continues to rise^1,2^. Fat accumulation in the visceral adipose tissue and liver has been shown to increase the secretion of proinflammatory cytokines, such as tumor necrosis factor (TNF)-α, interleukin (IL)-1, IL-6, and leptin, leading to metabolic tissue inflammation and insulin resistance in obese individuals^3–5^. Thus, obesity is a major risk factor for metabolic diseases, such as type 2 diabetes, nonalcoholic fatty liver disease, and cardiovascular disease^4,6,7^.

The gut microbiome has been shown to have essential roles in the synthesis of essential amino acids and vitamins, elimination of toxic compounds, and regulation of the immune system^8–11^. The composition of the gut microbiome is affected by various factors, such as genetics, geographical location, and age^12,13^, and dysbiosis or imbalance of the gut microbiome composition is correlated with inflammatory bowel diseases, cancer, neurological disorders, and metabolic diseases^14–16^. Obesity-related alterations in the gut microbiome composition are characterized by changes in the *Firmicutes* to *Bacteroidetes* ratio, which is increased in obese humans and mice^17,18^. Recently, several studies have reported that the administration of *Lactobacillus* spp. ameliorates diet-induced body weight gain and fat accumulation and decreases the levels of serum metabolic biomarkers, such as blood glucose, insulin, and triglycerides^19–22^. However, the underlying mechanisms through which *Lactobacillus* spp. ameliorated diet-induced obesity remain unclear.

Accordingly, in this study, we evaluated the protective effects of *Lactobacillus fermentum* LM1016 against diet-induced obesity. Our results provide important insights into the potential applications of *L. fermentum* LM1016 administration for the treatment of metabolic disorders, including obesity.

## Research design and methods

### Mouse models

Animal procedures were approved by the Institutional Animal Care and Use Committee of CHA University. Male C57BL/6N mice (6 weeks old) were obtained from Orient Bio (Gapyeong, Gyeonggi, Korea). One week after adaptation, the mice were fed a normal diet (ND; 2018; Envigo, Indianapolis, IN, USA) with phosphate-buffered saline (PBS), a high-fat diet (HFD; 60% of calories from fat; D12492; Research Diets, New Brunswick, NJ, USA) with PBS, or a HFD with probiotics. Body weights were measured once a week. After 8 weeks, mice were sacrificed in a CO_2_ chamber. Blood was collected by cardiac puncture after CO_2_ sacrifice. Liver and adipose tissues (gonadal fat, inguinal fat, and brown adipose tissue) were dissected precisely and weighed.

### Bacterial administration

*L. fermentum* LM1016 was obtained from Lactomason (South Korea). *L. acidophilus* Rosell418 was obtained from Lallemand (Canada). *L. acidophilus* La14, *L. salvarius* Lrs-33, *L. rhamnosus* HOWARU Rhamnosus, and *L. paracasei* LPC37 were obtained from DuPont (USA). *L. helveticus* LH166 was obtained from Culture System Inc. (USA). Each *Lactobacillus* strain was resuspended in PBS, and mice were orally administered 1 × 10^9^ CFU of bacteria once a day for 8 weeks.

### Colonization of *Lactobacillus fermentum* LM1016

For colonization experiments, mice were orally administered 1 × 10^9^ CFU of bacteria once, and stool samples were collected 4, 8, 12, and 24 h after administration. DNA extractions were performed on 500 mg of feces per sample using the FastDNA SPIN Kit for Soil (MP Biomedicals, Solon, CA, USA). *L. fermentum* LM1016 abundance was determined using a quantitative real-time PCR (qPCR) assay with *L. fermentum*-specific primers^23^ and normalized to 16S rRNA-specific primers^24^. TaqMan™ Fast Advanced Master Mix (Applied Biosystems, Foster City, CA, USA) and the StepOnePlus real-time PCR system (Applied Biosystems) were used for qPCR experiments. The primer sequences are listed in Supplementary Table 1.

### Tissue histology

Liver, brown adipose tissue (BAT), and gonadal white adipose tissue (gWAT) were fixed in 4% paraformaldehyde for 24 h. The tissues were then sequentially dehydrated in increasing concentrations of ethanol (50–100%). Dehydrated tissue was infiltrated with 100% xylene, embedded in paraffin, and sectioned (4 μm). Hematoxylin and eosin (H&E) staining was performed according to standard methods.

### Liver triglyceride levels

Liver extracts were prepared and incubated in a buffer containing 5% NP-40/ddH_2_O solution, and supernatants containing triglycerides were separated. Triglyceride concentrations were measured on the supernatant fraction via a commercial colorimetric assay kit according to the manufacturer’s recommendations (Abcam, Paris, France).

### RNA isolation and real-time polymerase chain reaction (PCR)

Total RNA was extracted using TRIzol reagent according to the manufacturer’s instructions. The purity and concentration of total RNA were determined using a NanoDrop One Spectrophotometer (Thermo Fisher). Briefly, 1 μg of total RNA was reverse transcribed using a PrimeScript 1st strand cDNA synthesis kit (TaKaRa, Japan). Real-time PCR was performed using a CFX384 Touch real-time PCR System (Bio-Rad, Hercules, CA, USA) with SYBR Premix Ex Taq (TaKaRa) and gene-specific primers. The thermal cycling conditions were as follows: 95 °C for 10 min, followed by 35 cycles of 95 °C for 10 s, 60 °C for 15 s, and 72 °C for 15 s. The comparative threshold cycle (CT) was used to analyze the relative changes in gene expression normalized to *36B4* mRNA expression.

### Biochemical analysis of mouse serum

Serum insulin levels were measured in a 96-well microplate using a commercial Morinaga Ultra Sensitive Mouse/Rat Insulin ELISA Kit (MIoBS, Japan). The absorbance of each well was determined at 450 nm with a microplate reader (Molecular Device, CA, USA). Serum leptin levels were measured in a 96-well microplate using a commercial Morinaga Mouse/Rat Leptin ELISA Kit (MIoBS), with absorbance measurements at 450 nm. Serum total cholesterol levels were measured using an automated apparatus (Mindray BS-200; Shenzhen, China). Serum bile acid levels were measured using a spectrophotometric enzymatic assay (Sigma Diagnostics, Inc., St. Louis, MO, USA) according to the manufacturer’s instructions. Serum C-reactive protein (CRP) levels were measured using a mouse C-Reactive Protein/CRP Quantikine ELISA kit (R&D system, Minneapolis, MN) according to the manufacturer’s instructions. Glucose tolerance tests (GTTs) were performed after 12 h of fasting. Mice were injected with 2 g glucose per kg body weight intraperitoneally, and glucose concentrations were measured at 0, 15, 30, 60, and 120 min after injection.

### Transcriptome sequencing and gene set enrichment analysis (GSEA)

RNA extraction from mouse intestines was performed using an RNeasy Mini Kit (Qiagen, Valencia, CA, USA). The 101-bp paired-end libraries were constructed with a TruSeq RNA Sample Prep Kit v2 (Illumina, San Diego, CA, USA) using 1 μg of RNA. Whole-transcriptome sequencing (WTS) was performed on an Illumina HiSeq 2500 instrument. RNA-seq reads from each WTS experiment were aligned to the mouse reference genome (GRCm38) using STAR aligner^25^. Gene expression levels were quantified using RSEM^26^. To identify gene sets enriched in samples showing synergistic effects compared with samples without synergistic effects, GSEA was performed using javaGSEA desktop application (GSEA v2.1.0)^27,28^. Gene sets from gene ontology (GO) biological processes were used, and gene sets containing fewer than 15 genes and more than 500 genes were excluded. The *p*-values were calculated by permuting the data 1000 times for finding enriched gene sets. The GSEA software produced an enrichment score (ES), normalized ES, nominal *p*-value, and false discovery rate (*Q*-value). Gene sets that were up- or downregulated with a *Q*-value of less than 0.25 were considered significant.

### Metabolomics profiling of mouse plasma, MRS medium, and bacterial supernatants

#### Ultra-performance liquid chromatography/quadrupole time-of-flight mass spectrometry (UPLC-Q-TOF/MS) analysis

Four volumes of 80% methanol in water were added to 20 μL of mouse plasma, bacterial culture supernatants, or MRS media. After vortexing for 1 h at room temperature, the mixtures were centrifuged at 13,000×*g* for 20 min at 4 °C. The supernatants were transferred to sample vials and analyzed by UPLC-Q-TOF/MS (Synapt G2Si; Waters, USA). UPLC separation was performed using an Acquity UPLC BEH C18 column (2.1 mm × 100 mm, 1.7 μm; Waters). Mobile phase A consisted of 0.1% formic acid in the water, whereas mobile phase B contained 0.1% formic acid acetonitrile. Samples were eluted using the following conditions: 0% B increased to 5% B at 1 min, increased to 95% B at 9.0 min, increased to 100% B at 10.5 min, decreased to 0% B at 11 min, and equilibrated for an additional 1.5 min. The flow rate was 0.4 mL/min. The column temperature was maintained at 40 °C. The mass acquisition was performed in both positive (ESI+) and negative (ESI−) electrospray ionization modes with the following parameters: capillary voltage of 2.0 kV; cone voltage of 10 V; source temperature of 110 °C; desolvation temperature of 400 °C; and desolvation gas flow of 650 L/h. Mass data were collected in the range of *m*/*z* 60–1400 with a scan time of 0.25 s and an interscan time of 0.02 s for 12 min. Mass data, including the retention time, *m*/*z*, and ion intensities, were extracted using Progenesis QI software (Waters).

For metabolite identification, the following resources were used: exact mass, molecular formula suggested by the MassLynx software (Waters) based on the element composition and isotope composition of the parent mass ion; MS/MS spectra of the compounds; and metabolome databases, including the Human Metabolomics Database (http://www.hmdb.ca/), METLIN (http://metlin.scripps.edu/), MASS Bank (http://www.massbank.jp/), and LIPID MAPS (http://www.lipidmaps.org/).

### Bacterial transcriptome sequencing and annotation

Bacterial RNA was extracted using a ZymoBIOMICS RNA Miniprep Kit (Zymo Research). Sequencing and library construction were performed on an Illumina HiSeq 2500 with 101-bp paired-ends. Ribosomal RNA was removed using a Ribo-Zero rRNA Removal Kit (Bacteria) (Epicentre). Libraries were prepared with a TruSeq RNA Sample Prep kit v2 (Illumina). RNA-sequenced reads were mapped to the *L. fermentum* genome (NCBI, NC010610.1) using STAR with alignIntron MAX 1^25^. Then, the mapped reads were used to calculate read counts of genes using cufflinks^29^, and the gene list was input into the Cytoscape plug-in ClueGO v2.5.4^30^ to annotate functionally grouped networks. Functionally related GO terms for biological processes in *Escherichia coli* (version: 18 November 2016) were grouped based on a kappa score greater than 0.4 with a network specificity of 4–10. Statistical significance was calculated using two-sided hypergeometric tests, and the false discovery rate was corrected using the Bonferroni step down method.

### Preparation of bacterial extract

*L. fermentum* LM1016 was harvested in MRS media and washed twice with PBS. *L. fermentum* LM1016 cells (1 × 10^9^ CFU/mL) were lysed using FastPrep-24 5 G (MP Biomedicals, USA) and centrifuged at 15,000 rpm for 5 min. The supernatants were used after filtration with a 0.2-μm filter.

### Differentiation of 3T3-L1 preadipocytes and cell culture

3T3-L1 preadipocytes were cultured in vitro in Dulbecco’s modified Eagle’s medium (DMEM high glucose; Gibco) containing 10% bovine calf serum (Gibco) and 1% penicillin/streptomycin (Gibco) in an atmosphere of 95% humidified air/5% CO_2_. Cells were passaged prior to reaching confluence. Next, cells were seeded at a density of 4 × 10^5^ cells/well in a 6-well plate and allowed to reach 100% confluence (day 0). The basal medium was then replaced with DMEM high glucose containing 10% fetal bovine serum (FBS; Gibco), 1% penicillin/streptomycin, 0.5 mM IBMX, 1 μM dexamethasone, and 10 μg/mL insulin solution. On day 2, the medium was changed to DMEM containing 10% FBS, 1% penicillin/streptomycin, and 1% bacterial extract or 10 μg/mL linoleic acid (Merck, Germany). The media were changed every 2 days. On day 10, 3T3-L1 adipocyte differentiation was observed by Oil Red O staining. RAW 264.7 macrophage cells were obtained from the Korean Cell Line Bank (Seoul, Korean) and cultured in DMEM containing 10% bovine calf serum (Gibco) and 1% penicillin/streptomycin (Gibco) at 37 °C in an atmosphere of 95% humidified air/5% CO_2_.

### Oil Red O staining

3T3-L1 cells were washed with PBS twice, fixed with 10% formalin at room temperature for 10 min, and stained with Oil Red O (Sigma) at 37 °C for 10 min.

### Protein levels of IL-6 and TNF-α in the culture medium

Cells were treated with 100 ng/mL lipopolysaccharide (LPS; Sigma-Aldrich) with or without 1% bacterial extraction medium for 24 h. IL-6 and TNF-α concentrations were measured using ELISA kits.

### Statistical analysis

In vivo and in vitro data were analyzed using Prism 4.0 (GraphPad). Body weights and GTTs were evaluated by two-way analysis of variance (ANOVA). Statistical analysis of different groups was performed using Student’s *t*-test or one-way ANOVA.

## Results

### Administration of *L. fermentum* reduced diet-induced body weight gain

**Fig. 1:**
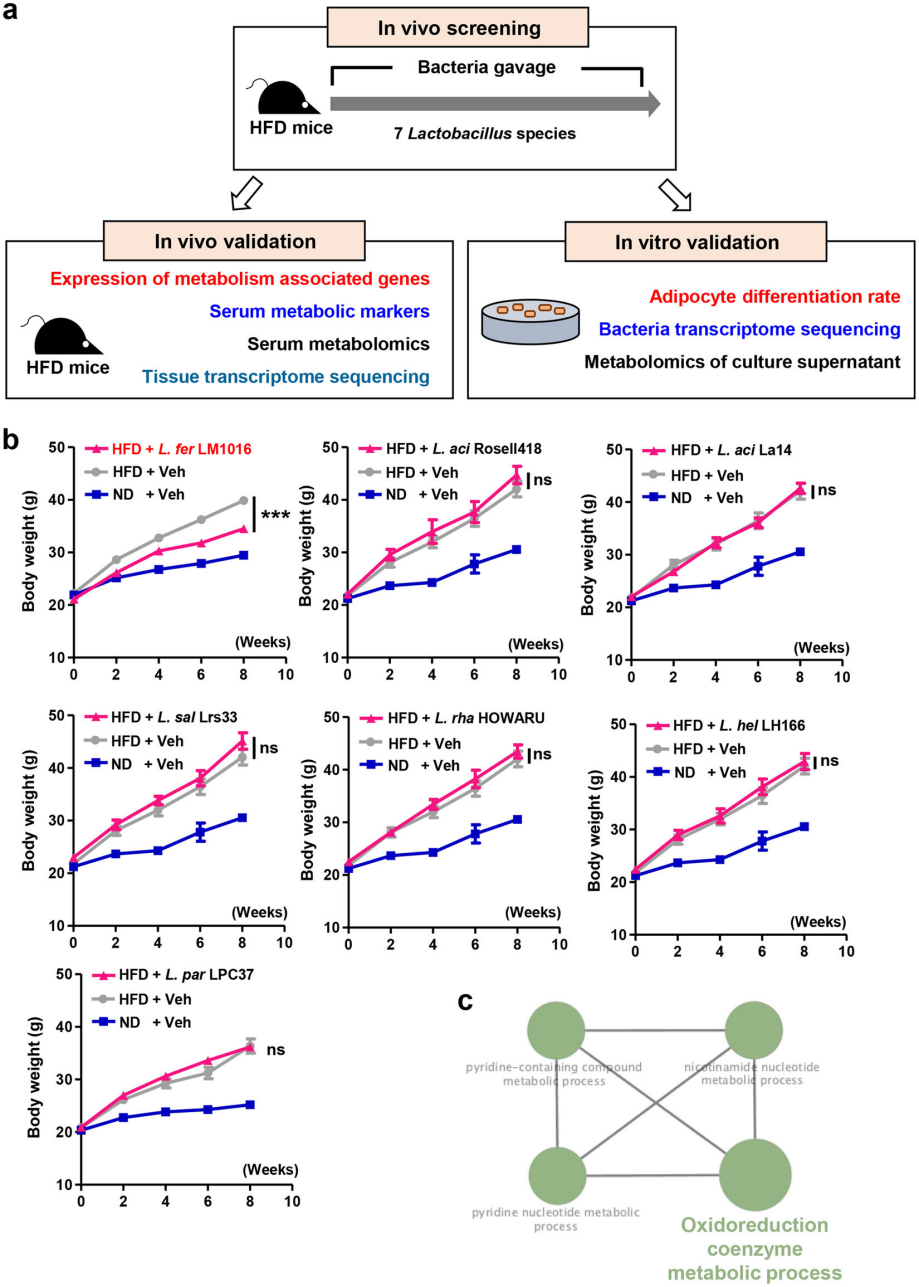
Administration of *L. fermentum* LM1016 reduced diet-induced obesity. **a** Schematic representation of the in vivo and in vitro experiments. **b** Body weight curves in normal diet (ND)- and high-fat diet (HFD)-fed mice and HFD-fed mice treated with *Lactobacillus* strains. **c** The ClueGO functionally grouped network of *L. fermentum* LM1016. ns not significant, ***p < 0.001, as determined by two-way ANOVA (**b**). Data are expressed as means ± SEMs.

To identify the strains of *Lactobacillus* spp. that protect against diet-induced obesity, we tested seven *Lactobacillus* strains (Supplementary Table S2) in HFD-fed mice (Fig. 1a). Although HFD-fed mice showed significant increases in body weight gain compared with ND-fed controls, we found that administration of *L. fermentum* LM1016 significantly reduced diet-induced body weight gain (Fig. 1b). Surprisingly, the administration of other strains of *Lactobacillus* did not have anti-obesity effects in HFD-fed mice (Fig. 1b). To examine the molecular mechanisms mediating the anti-obesity effects of *L. fermentum* LM1016, we performed WTS of *L. fermentum* LM1016. Several metabolic pathways, including the oxidoreduction coenzyme metabolic process, pyridine-containing compound metabolic process, nicotinamide nucleotide metabolic process, and pyridine nucleotide metabolic process, were identified as functionally grouped networks of *L. fermentum* LM1016 GO categories (Fig. 1c). The balance of oxidative stress and oxidoreduction has been shown to have crucial roles in the pathogenesis of metabolic syndromes, such as cardiovascular diseases, insulin resistance, and metabolic disorders^31–34^. Therefore, the functional gene network of the oxidoreduction coenzyme metabolic process was one of the key molecular mechanisms mediating the anti-obesity effects of *L. fermentum* LM1016. To determine whether *L. fermentum* LM1016 colonized the host intestinal tract after oral administration, we next harvested fecal samples at different time points after administration of *L. fermentum* LM1016. We observed that *L. fermentum* was localized for several hours after oral administration (Supplementary Fig. S1). However, *L. fermentum* was not detected in the fecal samples 24 h after oral administration, implying that *L. fermentum* transiently localized in the host intestinal tract (Supplementary Fig. S1). These data clearly suggest that daily treatment of *L. fermentum* LM1016 is required to modulate host metabolic homeostasis.

### Administration of *L. fermentum*-stimulated brown adipocyte function by increasing bile acid signaling

**Fig. 2:**
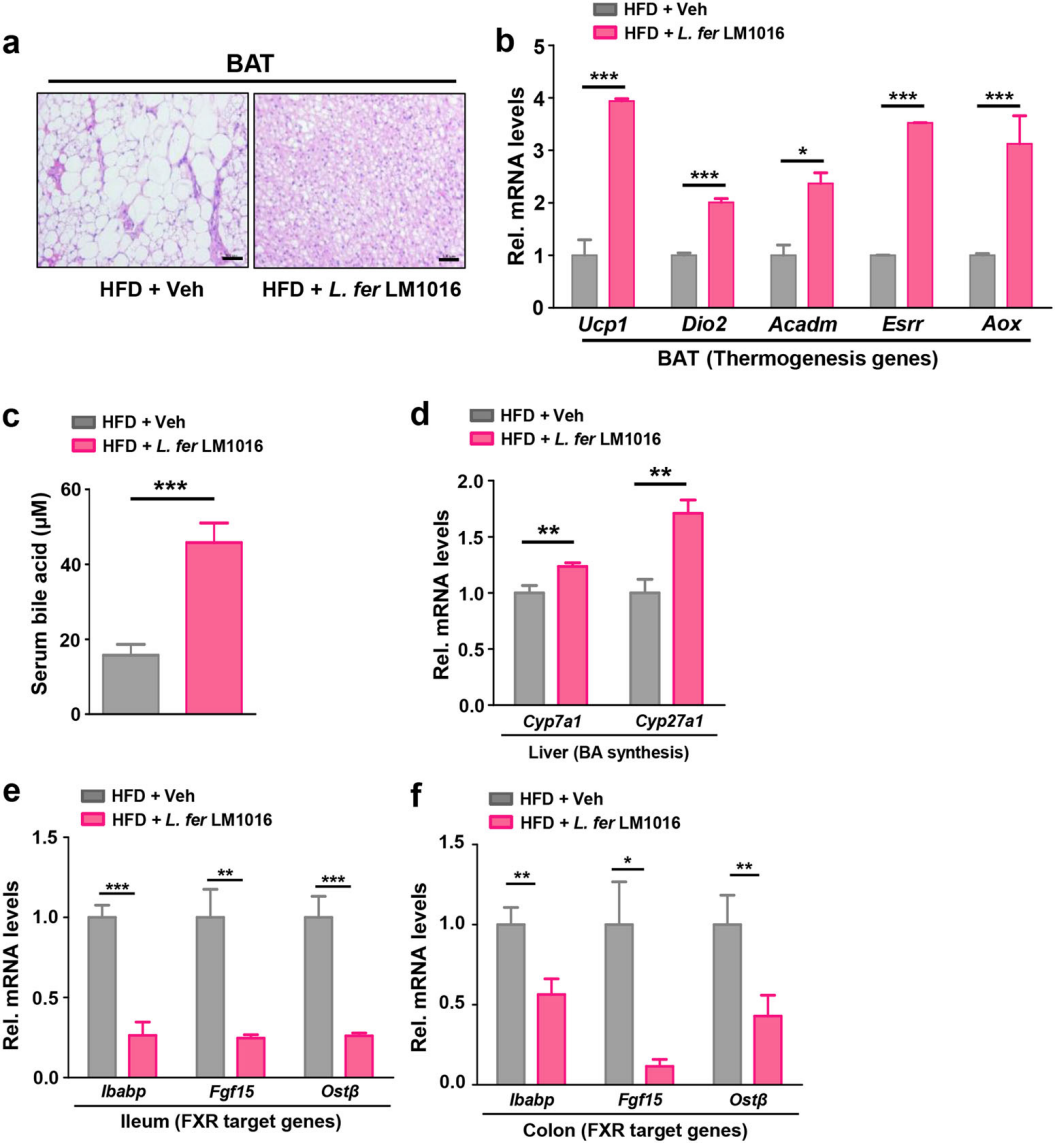
Administration of *L. fermentum-* stimulated brown adipocyte function by increasing serum levels of bile acids. **a** Hematoxylin–eosin (H&E) staining of brown adipose tissue (BAT). Scale bar, 100 μm. **b** mRNA expression of thermogenesis genes in BAT. **c** Serum bile acid levels. **d** mRNA expression of bile acid (BA) synthesis genes in the liver. **e** mRNA expression of FXR target genes in the ileum. **f** mRNA expression of FXR target genes in the colon. *p < 0.05, **p < 0.01, ***p < 0.001, as determined by Student’s *t*-test (**b**–**f**). Data are expressed as means ± SEMs.

Because *L. fermentum* LM1016 treatment markedly reduced body weight gain in HFD-fed mice, we next examined the cellular functions of BAT, including thermogenesis. BAT has important roles in both basal and adaptive energy metabolism by nonshivering thermogenesis and promotes energy expenditure to improve insulin sensitivity and reduce body weight gain^35–37^. Histological analysis revealed that lipid droplet sizes in BAT were reduced in *L. fermentum* LM1016-treated mice compared with vehicle-treated mice (Fig. 2a). With gene expression profiling analysis, we observed that the expression levels of genes involved in thermogenesis, including *Ucp1*, *Dio2*, *Acadm*, *Esrr*, and *Aox*, were significantly increased in BAT from *L. fermentum* LM1016-treated mice, suggesting that BAT-mediated energy expenditure was stimulated by administration of *L. fermentum* LM1016 (Fig. 2b).

Previous reports have shown that bile acids bind to the G protein-coupled receptor TGR5 to potentiate thyroid hormone signaling in BAT, leading to increased energy expenditure and reduced body weight gain^38^. We next determined whether administration of *L. fermentum* LM1016 increased bile acid signaling to potentiate BAT-mediated energy expenditure. Notably, serum bile acid levels were significantly elevated in *L. fermentum* LM1016-treated mice compared with vehicle-treated HFD mice (Fig. 2c). Bile acids are synthesized from cholesterol in hepatocytes by various liver enzymes, including cholesterol 7α-hydroxylase (CYP7A1), sterol 12α-hydroxylase, and sterol-27-hydroxylase (CYP27A1)^39^. Therefore, we next examined the expression levels of these genes in the liver. The expression of bile acid synthesis genes (i.e., *CYP7A1* and *CYP27A1*) was significantly upregulated in the livers of *L. fermentum* LM1016-treated mice compared with that in HFD mice, implying that *L. fermentum* LM1016 treatment increased hepatic bile acid synthesis (Fig. 2d). Previously, intestinal fibroblast growth factor 15 (FGF15) was shown to suppress hepatic bile acid synthesis by blocking CYP7A1 expression in mice^40^. In this study, we observed that *L. fermentum* LM1016 treatment markedly suppressed *FGF15* gene expression in the ileum and colon (Fig. 2e, f), suggesting that *L. fermentum* LM1016 treatment may modulate intestinal signaling to increase hepatic bile acid synthesis to stimulate BAT-mediated energy expenditure.

### Administration of *L. fermentum* improved serum metabolic parameters

**Fig. 3:**
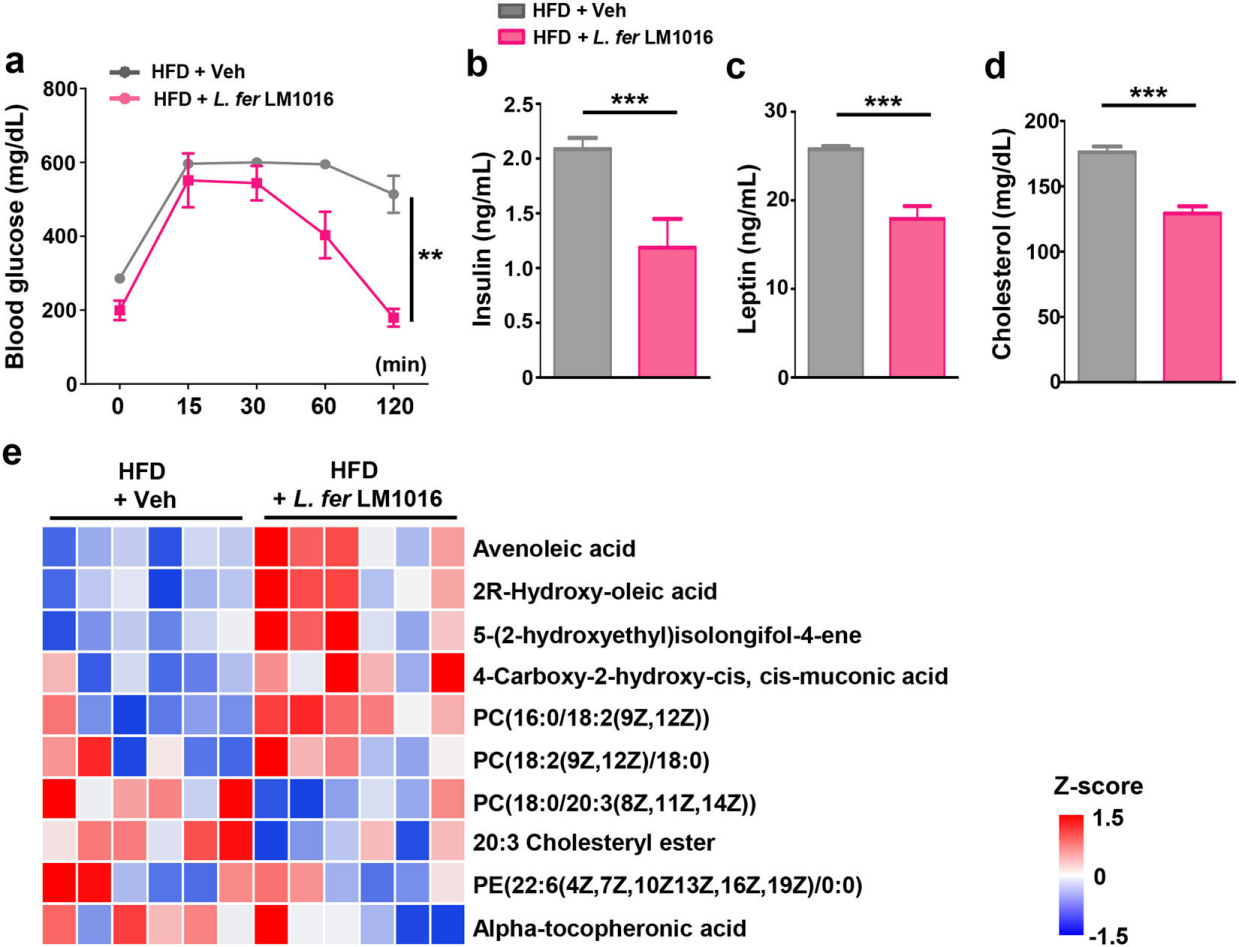
Administration of *L. fermentum* improved serum metabolic parameters. **a** Glucose tolerance test of HFD-fed mice treated with vehicle or *L. fermentum* LM1016. **b** Serum insulin levels. **c** Serum leptin levels. **d** Serum cholesterol levels. **e** Heatmap of differentially detected serum metabolites between high-fat diet (HFD)-fed mice and HFD-fed mice treated with *L. fermentum* LM1016. **p < 0.01, ***p < 0.001, as determined by two-way ANOVA (**a**) and Student’s *t*-test (**b**–**d**). Data are expressed as means ± SEMs.

Given that obesity is closely related to type 2 diabetes mellitus and metabolic disorders, we next examined whether administration of *L. fermentum* LM1016 improved serum metabolic biomarkers in diet-induced obese mice. GTT analysis revealed that fasting blood glucose levels were markedly reduced in *L. fermentum* LM1016-treated mice compared with vehicle-treated HFD-fed mice (Fig. 3a). While blood glucose levels were highly elevated after glucose injection in vehicle-treated HFD-fed mice, *L. fermentum* LM1016 treatment potentiated glucose clearance to reduce blood glucose levels (Fig. 3a). Consistent with the improvement in glucose clearance, we also observed that fasting serum insulin levels were markedly decreased by *L. fermentum* LM1016 treatment (Fig. 3b). Notably, blood levels of leptin and cholesterol were also significantly reduced in *L. fermentum* LM1016-treated mice (Fig. 3c, d).

The gut microbiome has been shown to produce diverse serum metabolites that regulate both metabolic homeostasis and immunity^41^. To explore the correlations between serum metabolites and *L. fermentum* LM1016 administration, we analyzed serum metabolomic profiling of vehicle-treated and *L. fermentum* LM1016-treated HFD-fed mice. We observed that several metabolites, including avenoleic acid, 2R-hydroxy-oleic acid, and PC (16:0/18:2(9Z,12Z)), were markedly increased by *L. fermentum* LM1016 administration (Fig. 3e). In prior studies, serum cholesteryl ester has been reported to reflect adiposity and to be associated with the insulin sensitivity index^42^. Similarly, in this study, we also observed that serum levels of 20:3 cholesteryl ester were markedly reduced in mice administered *L. fermentum* LM1016. Thus, the metabolomic analysis revealed that administration of *L. fermentum* LM1016 altered serum metabolites, resulting in modulation of metabolic homeostasis to reduce adiposity.

### Administration of *L. fermentum* reduced hepatic gluconeogenesis and lipogenesis to ameliorate hepatic steatosis

**Fig. 4:**
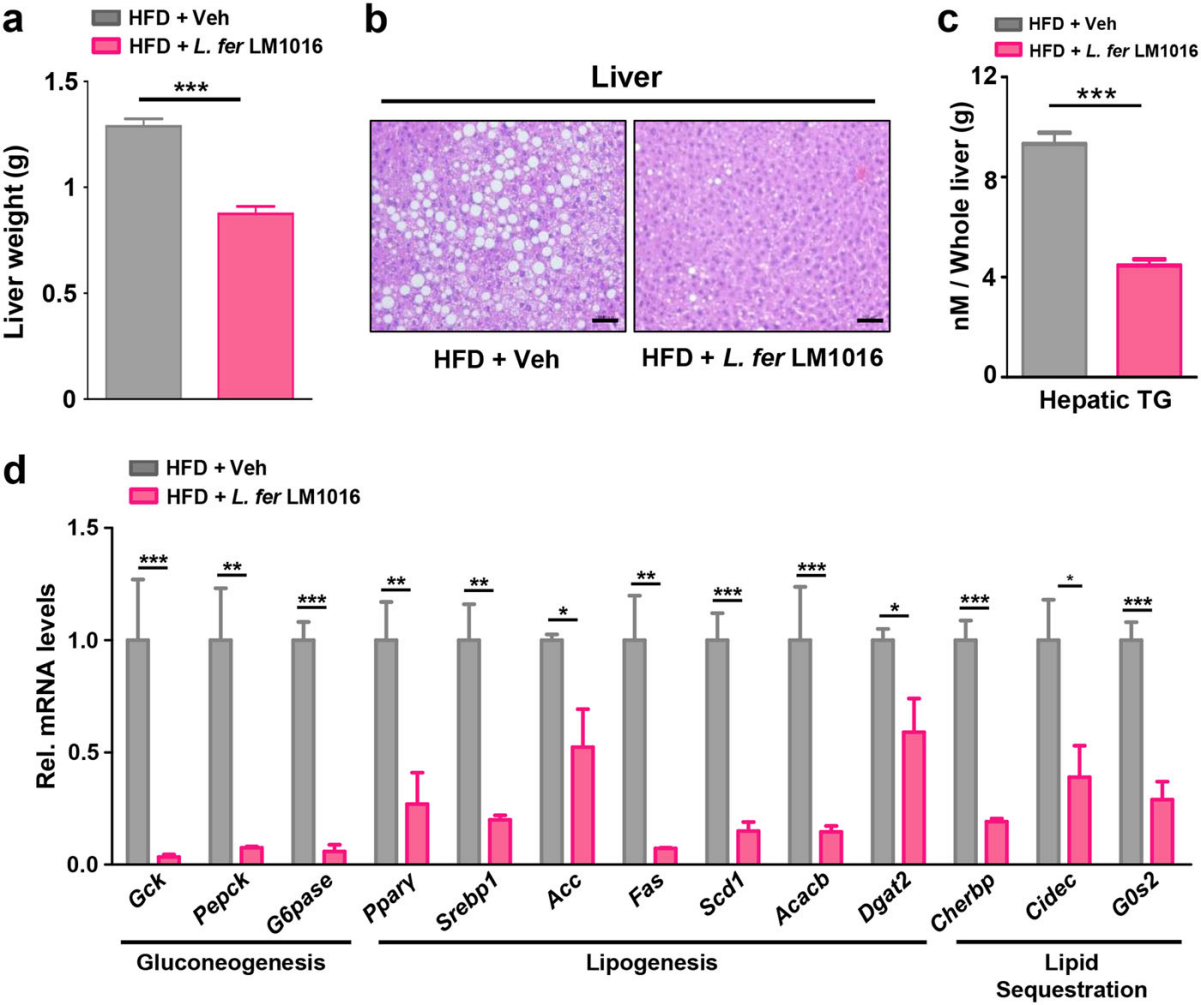
Administration of *L. fermentum* reduced hepatic gluconeogenesis and lipogenesis to ameliorate hepatic steatosis. **a** Liver weight. **b** Hematoxylin–eosin (H&E) staining of liver tissues. Scale bar, 100 μm. **c** Hepatic triglyceride concentrations. **d** mRNA expression of gluconeogenesis, lipogenesis, and lipid sequestration genes in the liver. *p < 0.05, **p < 0.01, ***p < 0.001, as determined by Student’s *t*-test (**a**, **c**, **d**). Data are expressed as the means ± SEMs.

We next examined the wet weights of livers from HFD-fed mice treated with vehicle or *L. fermentum* LM1016. We observed that liver weights were markedly reduced in HFD-fed mice treated with *L. fermentum* LM1016 (Fig. 4a). Hepatic steatosis, a well-known complication of obesity, is a condition of triglyceride accumulation in hepatocytes and can lead to hepatocyte injury, cell death, and fibrosis^43,44^. Administration of *L. fermentum* LM1016 led to a significant reduction in lipid droplet size and triglyceride accumulation in the liver (Fig. 4b, c). To investigate the molecular mechanisms of how *L. fermentum* LM1016-attenuated hepatic steatosis, we performed quantitative real-time PCR (qPCR) to confirm the expression of hepatic steatosis-associated genes. Hepatic genes associated with gluconeogenesis, lipogenesis, and lipid sequestration were markedly decreased in mice treated with *L. fermentum* LM1016 compared with those in hepatocytes from HFD-fed mice (Fig. 4d), implying that *L. fermentum* LM1016 modulated the expression levels of hepatic genes involved in metabolic pathways in the liver to improve liver physiology.

### Administration of *L. fermentum* promoted oxidative phosphorylation and attenuated inflammation in adipose tissue

**Fig. 5:**
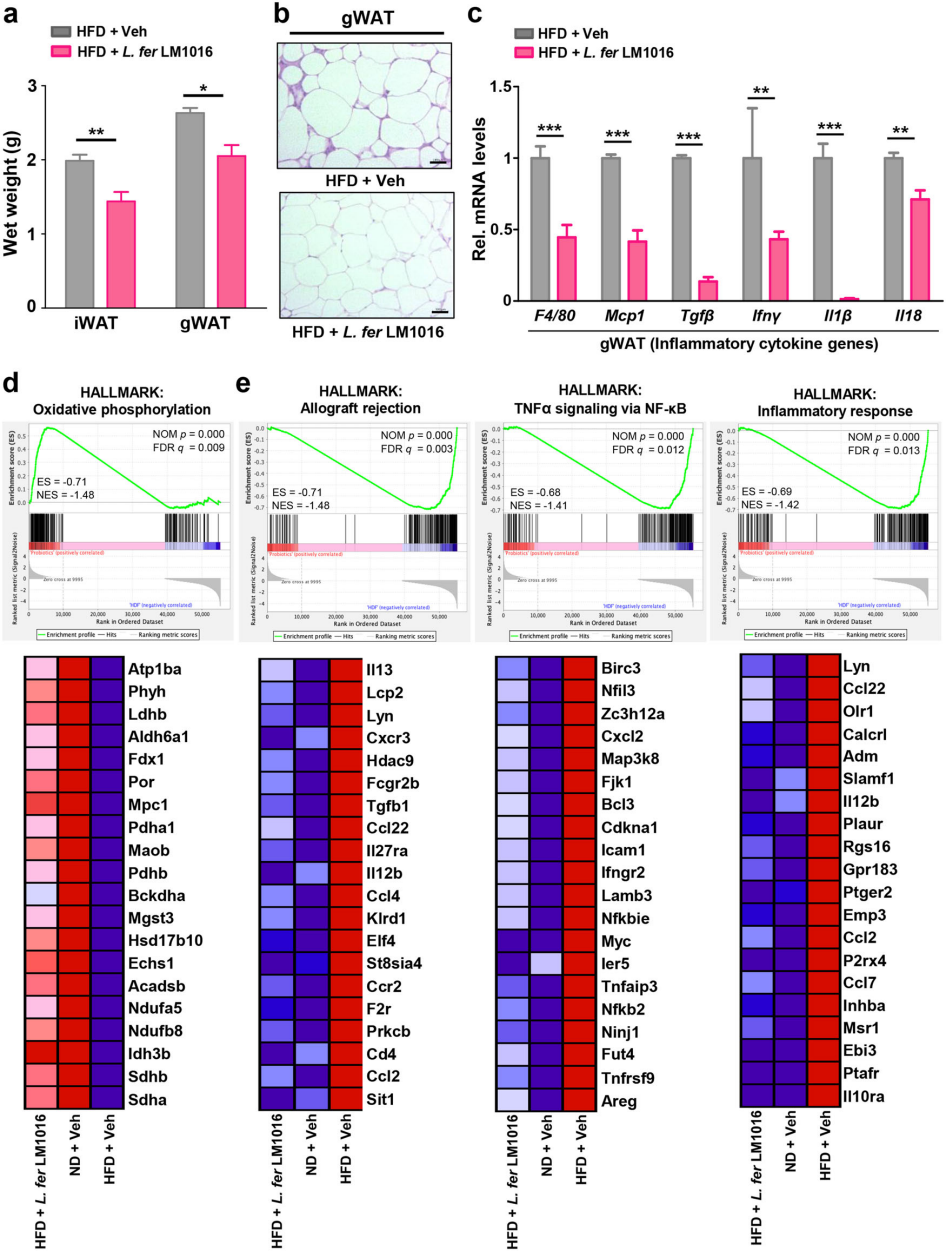
Administration of *L. fermentum* promoted oxidative phosphorylation and attenuated inflammation in adipose tissue. **a** Inguinal white adipose tissue (iWAT) and gonadal white adipose tissue (gWAT) weights. **b** Hematoxylin–eosin (H&E) staining of gWAT. Scale bar, 100 μm. **c** mRNA expression of inflammatory cytokine genes in gWAT. **d**, **e** Gene set enrichment analysis (GSEA) of representative significantly enriched hallmark signatures. ES enrichment score, NES net enrichment score, NOM p nominal *p*-value, *p < 0.05, **p < 0.01, ***p < 0.001, as determined by Student’s *t*-test (**a**, **c**). Data are expressed as means ± SEMs.

Consistent with the observed reductions in liver weight, we also found that *L. fermentum* LM1016 administration reduced the wet weights of inguinal (iWAT) and gonadal WAT (gWAT) (Fig. 5a). Histological analysis of gWAT showed a marked decrease in adipocyte size in mice treated with *L. fermentum* LM1016 compared with that in HFD-fed mice (Fig. 5b).

Obesity is associated with chronic adipose tissue inflammation, which is characterized by elevation of inflammatory cytokines, such as IL-18, TNF-α, and other adipokines released from adipose tissues^45,46^. Therefore, to determine whether the administration of *L. fermentum* LM1016-attenuated inflammation in adipose tissue, we examined the expression levels of inflammatory cytokine genes by qPCR. The results showed that the expression levels of *F4/80*, *Mcp1*, *TGF-β*, *IFN-γ*, *IL-1β*, and *IL-18* were significantly decreased in gWAT of *L. fermentum* LM1016-treated HFD-fed mice (Fig. 5c). To determine whether systemic inflammation was reduced, we further examined C-reactive protein (CRP), a nonspecific marker of systemic inflammation^47,48^. Consistent with the reduction in inflammation in adipose tissue, we observed that serum CRP levels were markedly reduced by oral treatment with *L. fermentum* LM1016, implying that *L. fermentum* LM1016 treatment largely ameliorated systemic inflammation (Supplementary Fig. S2).

Next, we performed WTS of gWAT to further validate the molecular mechanisms in *L. fermentum* LM1016-treated HFD-fed mice, vehicle-treated HFD-fed mice, and ND-fed mice. GSEA demonstrated that gene sets involved in oxidative phosphorylation were significantly enriched and upregulated in *L. fermentum* LM1016-treated HFD-fed mice and ND-fed mice compared with those in vehicle-treated HFD-fed mice (Fig. 5d). In contrast, gene sets including allograft rejection, TNF-α signaling via nuclear factor (NF)-κB, and inflammatory response were significantly downregulated in *L. fermentum* LM1016-treated HFD-fed mice and ND-fed mice, indicating that tissue inflammation was markedly downregulated in gonadal adipose tissue by *L. fermentum* LM1016 treatment in HFD-fed mice (Fig. 5e). To determine whether administration of *L. fermentum* LM1016 reduces inflammatory signaling in the intestinal tract, we further performed transcriptome analysis in colonic tissues. Consistent with gWAT transcriptome analysis, GSEA analysis revealed that the expression of genes involved in the inflammatory signaling pathway was markedly reduced by *L. fermentum* LM1016 administration in HFD-fed mice (Supplementary Fig. S3).

### Metabolomic profiling of bacterial culture supernatants revealed the anti-obesity metabolites of *L. fermentum*

**Fig. 6:**
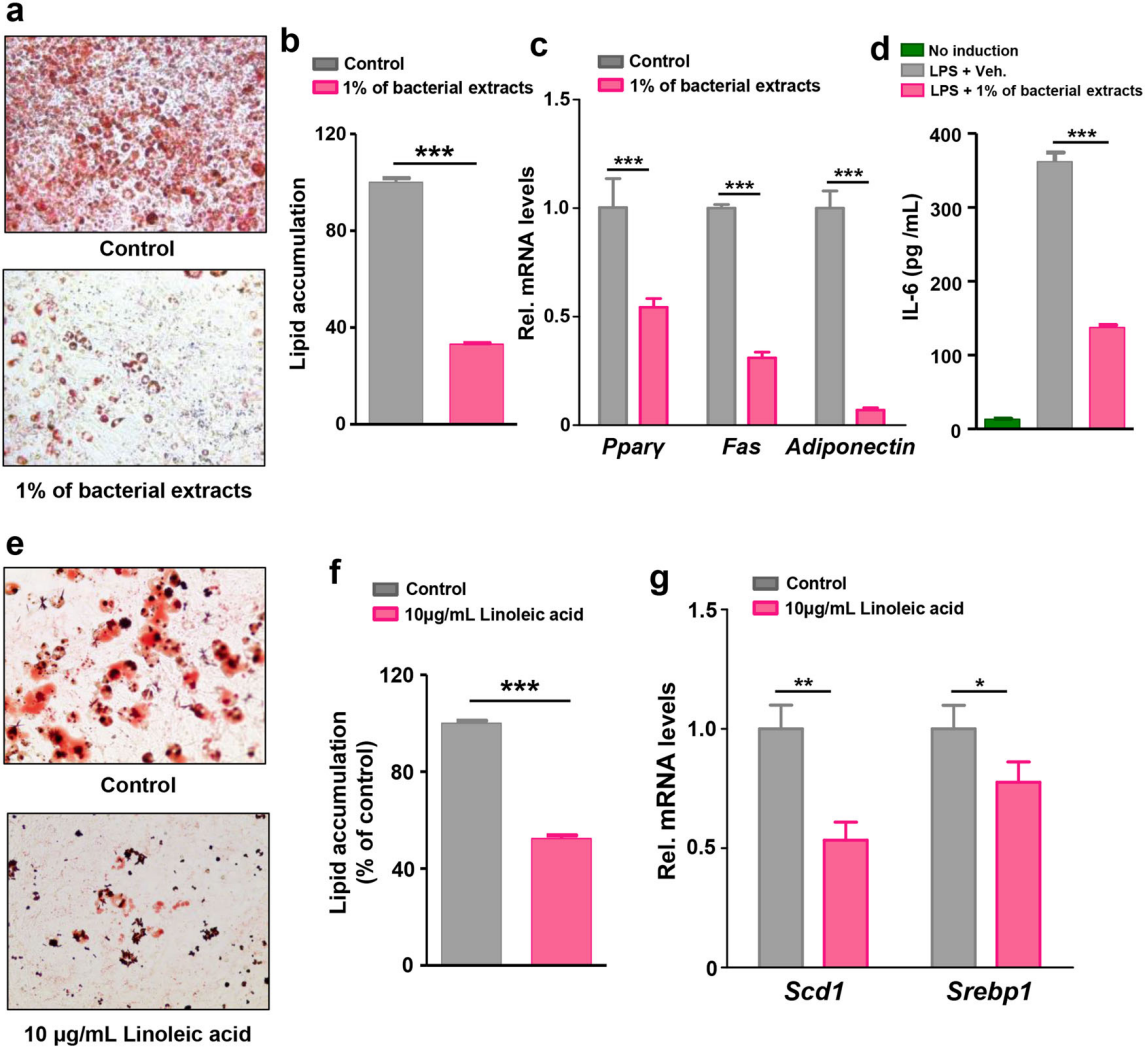
*L. fermentum LM1016*-derived metabolites reduced adipogenesis and inflammation in 3T3-L1 cells. **a**, **b** Effects of *L. fermentum* LM1016 bacterial extraction on 3T3-L1 cell differentiation. Lipid accumulation was determined by Oil Red O staining. **c** mRNA expression of adipogenesis genes in 3T3-L1 cells treated with or without bacterial extracts. **d** IL-6 concentrations in LPS-treated 3T3-L1 cells with or without bacterial extracts. **e**, **f** Effects of linoleic acid on 3T3-L1 cell differentiation. Lipid accumulation was determined by Oil Red O staining. **g** mRNA expression of adipogenesis genes in 3T3-L1 cells treated with or without linoleic acid. *p < 0.05, **p < 0.01, ***p < 0.001, as determined by Student’s *t*-test (**b**, **c**, **f**, **g**) or one-way ANOVA (**d**). Data are expressed as means ± SEMs.

To evaluate whether the gut microbiome controlled metabolic tissues, such as the liver and adipose tissue, we hypothesized that metabolites generated from *L. fermentum* LM1016 may have anti-obesity effects to reduce adipogenesis and lipid accumulation. We first tested 3T3-L1 preadipocyte differentiation following treatment with bacterial extracts from *L. fermentum* LM1016. Treatment with *L. fermentum* LM1016 bacterial extracts reduced the differentiation of mature adipocytes in 3T3-L1 cell cultures (Fig. 6a, b). Consistent with this, the expression levels of genes involved in adipogenesis, including *PPARγ*, *Fas*, and adiponectin, were downregulated by treatment with *L. fermentum* LM1016 bacterial extracts (Fig. 6c).

Previously, plasma levels of LPS were found to be positively correlated with insulin resistance, fasting hyperglycemia, and inflammation in adipose tissue^49^. Thus, we examined the anti-inflammatory effects of *L. fermentum* LM1016 bacterial extracts in LPS-treated 3T3-L1 preadipocytes. We treated cells with LPS (100 ng/mL) and *L. fermentum* LM1016 bacterial extracts for 24 h and then carried out enzyme-linked immunosorbent assays (ELISAs) to measure IL-6 in the cell culture medium. The results revealed that IL-6 concentrations were significantly decreased following treatment with LPS and *L. fermentum* LM1016 bacterial extracts compared with concentrations after treatment with LPS only (Fig. 6d).

To identify the metabolites of *L. fermentum* LM1016 that mediated its anti-obesity effects, we analyzed the metabolites in *L. fermentum* LM1016 culture supernatants and MRS medium. Metabolomic profiling revealed that 12 metabolites were only present in *L. fermentum* LM1016 culture supernatants and that these metabolites were derived from *L. fermentum* LM1016 (Table 1). Notably, linoleic acid was only present in *L. fermentum* LM1016 culture media (Table 1). This result reflected the serum metabolomics analysis in which *L. fermentum* LM1016 treatment elevated serum levels of avenoleic acid, a derivative of linoleic acid, in HFD-fed mice (Fig. 3e). To determine whether linoleic acid had anti-obesity properties, we examined the molecular mechanisms of how linoleic acid modulated adipogenesis in 3T3-L1 preadipocytes in vitro. We observed that mature adipocyte differentiation and lipid accumulation were markedly inhibited by linoleic acid treatment (Fig. 6e, f). Moreover, the expression levels of genes involved in adipogenesis, including *Scd1* and *Srebp1*, were also significantly decreased in 3T3-L1 cells treated with linoleic acid (Fig. 6g).

**Table 1:** Comparison of metabolite concentrations between bacterial culture supernatants and control MRS media.

	MRS media	Culture supernatant	*p-* value
Vaccenic acid	0 ± 0	6.86 ± 0.03	<0.001
Linoleic acid	0 ± 0	4.85 ± 0.18	<0.001
Palmitoleic acid ethyl ester	0 ± 0	6.85 ± 0.03	<0.001
Oxostearic acid	0 ± 0	6.24 ± 0.03	<0.001
Chaulmoogric acid	0 ± 0	6.14 ± 0.04	<0.001
8,9-Epoxyeicosatrienoic acid	0 ± 0	5.57 ± 0.02	<0.001
Indolelactic acid	0 ± 0	5.80 ± 0.05	<0.001
Ethyl pentadecanoate	0 ± 0	5.10 ± 0.10	<0.001
(*R*)-2-hydroxystearic acid	0 ± 0	7.91 ± 0.08	<0.001
[8-[2-(3-methylbutanoyloxy)propan-2-yl]-2-oxo-8,9-dihydrofuro[2,3-h]chromen-9-yl] (*Z*)-2-methylbut-2-enoate	0 ± 0	5.39 ± 0.06	<0.001
17.alpha.-Hydroxyethyl-5.beta.-estrane-3.alpha.,17.beta.-diol	0 ± 0	6.68 ± 0.06	< 0.001
[(2*S*,4As,6*R*)-3,4′-dihydroxy-1,1,4a,6-tetramethyl-6′-oxospiro[3,4,6,7,8,8a-hexahydro-2H-naphthalene-5,2′-7,8-dihydro-3H-furo[2,3-e]isoindole]-2-yl] acetate	0 ± 0	4.29 ± 0.47	0.004

Normalized log10.

Statistical analysis was determined by Student’s *t*-test, Data are expressed as means ± SEMs.

To examine whether both *L. fermentum* LM1016 bacterial extracts and linoleic acids were able to modulate inflammatory signaling pathways in immune cells, we next determined the gene expression of inflammatory cytokines in RAW 264.7 macrophage cells in the presence or absence of *L. fermentum* LM1016 bacterial extracts and linoleic acid. Consistently, we observed that treatment of *L. fermentum* LM1016 bacterial extracts and linoleic acid substantially repressed the expression levels of inflammatory cytokine genes, such as IL-1β, TNF-α, F4/80, and Mcp1, in RAW 264.7 macrophage cells (Supplementary Fig. S4a). We next measured the protein level of TNF-α. Consistently, treatment with both *L. fermentum* LM1016 bacterial extracts and linoleic acid markedly reduced the protein level of TNF-α secreted by RAW 264.7 macrophage cells (Supplementary Fig. S4b). Similar to RAW 264.7 macrophage cells, we also observed that treatment with both *L. fermentum* LM1016 bacterial extracts and linoleic acid markedly reduced the expression of inflammatory cytokine genes in 3T3-L1 cells (Supplementary Fig. S5). Altogether, these results demonstrated that *L. fermentum* LM1016 and its metabolites were able to reduce inflammatory signaling pathways in various tissues to protect against diet-induced obesity.

## Discussion

Environmental factors, such as diet, antibiotics, and surgery, are risk factors for dysbiosis and the development of metabolic disorders, including obesity^50–53^. Administration of several *Lactobacillus* strains has been shown to reduce abdominal adiposity, body weight, and body mass index in animal models and humans^20,52,54^. However, the anti-obesity mechanisms of *Lactobacillus* strains have not been fully demonstrated at the genetic and metabolomic levels. In this study, we found that administration of *L. fermentum* LM1016 suppressed diet-induced obesity and hepatic steatosis and improved serum metabolic markers, such as glucose, insulin, leptin, and cholesterol. Moreover, GSEA of gWAT revealed that genes involved in oxidative phosphorylation were enriched in gonadal adipose tissue of *L. fermentum* LM1016-treated mice, whereas genes involved in allograft rejection, TNF-α signaling via NF-κB, and inflammatory responses were markedly enriched in the gonadal adipose tissue of vehicle-treated mice. Given that obesity-associated metabolic disorders have been shown to correlate with tissue inflammation and oxidative stress^3,55–57^, administration of *L. fermentum* LM1016 may ameliorate oxidative stress and inflammation in WAT.

Bile acid synthesis is a complicated metabolic pathway involving catabolism of cholesterol by numerous hepatic cytochrome P450 enzymes, including CYP7A1 and CYP27A1^39,58^. Farnesoid X receptor (FXR) has been shown to regulate bile acid synthesis, and intestinal FXR activation suppresses bile acid synthesis via mouse FGF15, an ortholog of human FGF19^40^. In this study, we showed that administration of *L. fermentum* LM1016 suppressed the expression of FXR target genes, including *FGF15, Ibabp*, and *Ostβ*, in the ileum and colon (Fig. 2e, f), leading to induction of hepatic bile acid synthesis. Disruption of the gut microbiota, including reduced bile acid-metabolizing bacteria, markedly impairs bile acid metabolism, leading to dysregulation of host glucose and cholesterol homeostasis and alterations to the immune system^59^. Interestingly, *L. rhamnosus* GG has been reported to increase intestinal FXR/FGF15 signaling, leading to decreased hepatic bile acid synthesis and prevention of excessive bile acid-induced liver injury and fibrosis in mice^60^. These findings implied that each species of *Lactobacillus* may have its own physiological functions in regulating intestinal FXR/FGF15 signaling and thereby control hepatic bile acid synthesis in a species-dependent manner.

Using metabolomics analysis, we found that specific metabolites, including linoleic acid, vaccenic acid, and ethyl pentadecanoate, were only identified in the culture supernatants of *L. fermentum* LM1016. Previous reports showed that supplementation with vaccenic acid reduces serum levels of triglyceride, total cholesterol, and low-density lipoprotein (LDL) cholesterol^61^. Additionally, supplementation with conjugated linoleic acid reduced body weight gain and fat accumulation in animals and humans^62,63^. Because these metabolites are secreted or released from the bacterial wall component, these results suggested that administration of *L. fermentum* LM1016 may increase bacteria-derived specific metabolites in the blood, leading to decreased body weight gain in the HFD-fed mouse model. Furthermore, we observed that linoleic acid inhibited adipocyte differentiation and inflammatory responses in 3T3-L1 preadipocytes, suggesting that linoleic acid may be a potent anti-obesity metabolite derived from *L. fermentum* LM1016.

In summary, we demonstrated that administration of the gut microbe *L. fermentum* LM1016 ameliorated diet-induced obesity and improved metabolic biomarkers, including glucose, insulin, and leptin. Our results suggest that *L. fermentum* LM1016 treatment may be a potential therapeutic strategy for improving metabolic disorders, such as diet-induced obesity.

## Supplementary information

Supplementary information

## Data Availability

The sequencing data reported in this paper were deposited in the European Nucleotide Archive (accession no. ERP120924).
