# Supplementary material for: Lactobacillus fermentum promotes adipose tissue oxidative phosphorylation to protect against diet-induced obesity
Source: Exp Mol Med. 2020 Sep 11;52(9):1574–86. doi: 10.1038/s12276-020-00502-w (PMC8080655; doi:10.1038/s12276-020-00502-w)
Supplement: Supplementary file 1 — Supplementary information [file 12276_2020_502_MOESM1_ESM.pdf]

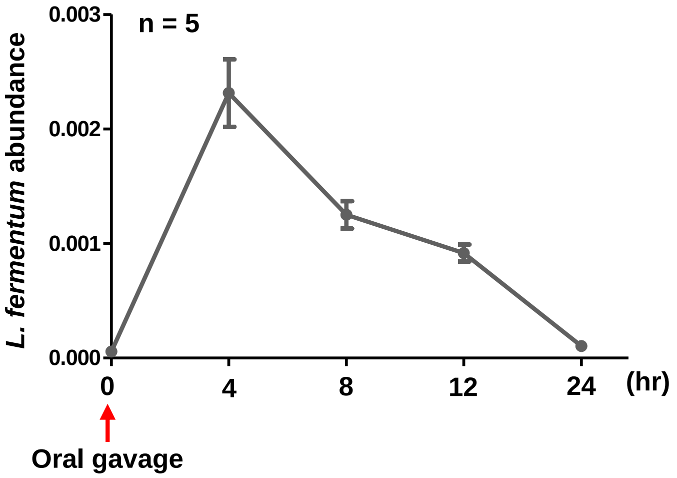

Supplementary Figure S1. *L. fermentum* LM1016 transiently localizes in host intestinal tract after oral administration. Mice were orally administered with L. fermentum LM1016 and their fecal samples were harvested at different time point to analyze L. fermentum abundance. Data are expressed as means ± SEMs.

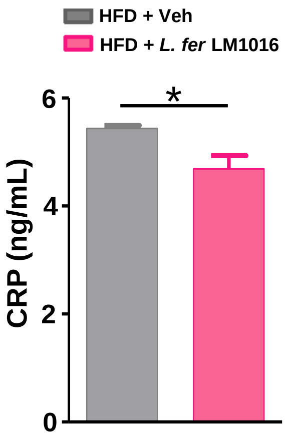

Supplementary Figure S2. *L. fermentum* LM1016 remarkably reduces systemic inflammation. Mice were orally administered with *L. fermentum* LM1016 and their serum samples were further analyzed to measure C-reactive protein, a non-specific marker for systemic inflammation. *$p < 0.05$ as determined by Student's t-test. Data are expressed as means ± SEMs.

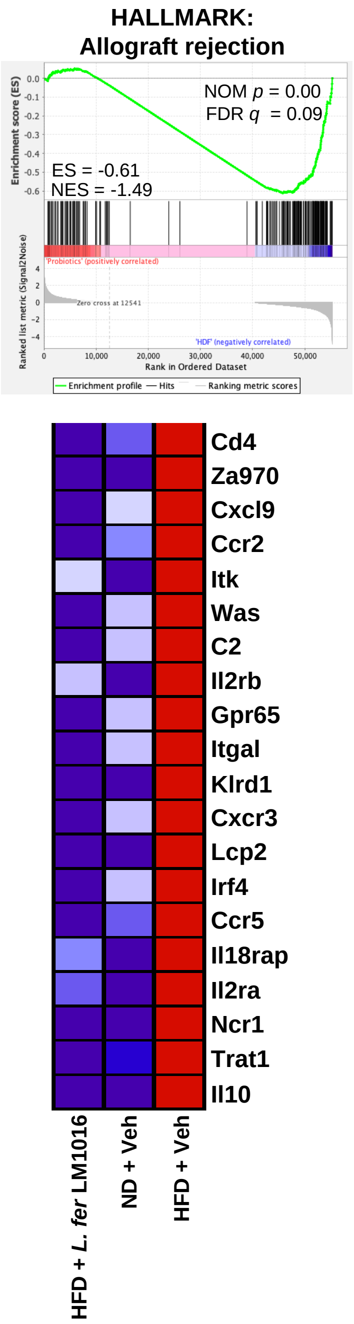

Supplementary Figure S3. *L. fermentum* LM1016 remarkably reduces inflammatory signaling pathway in colonic tissue. Gene set enrichment analysis (GSEA) of representative significantly enriched hallmark signatures.

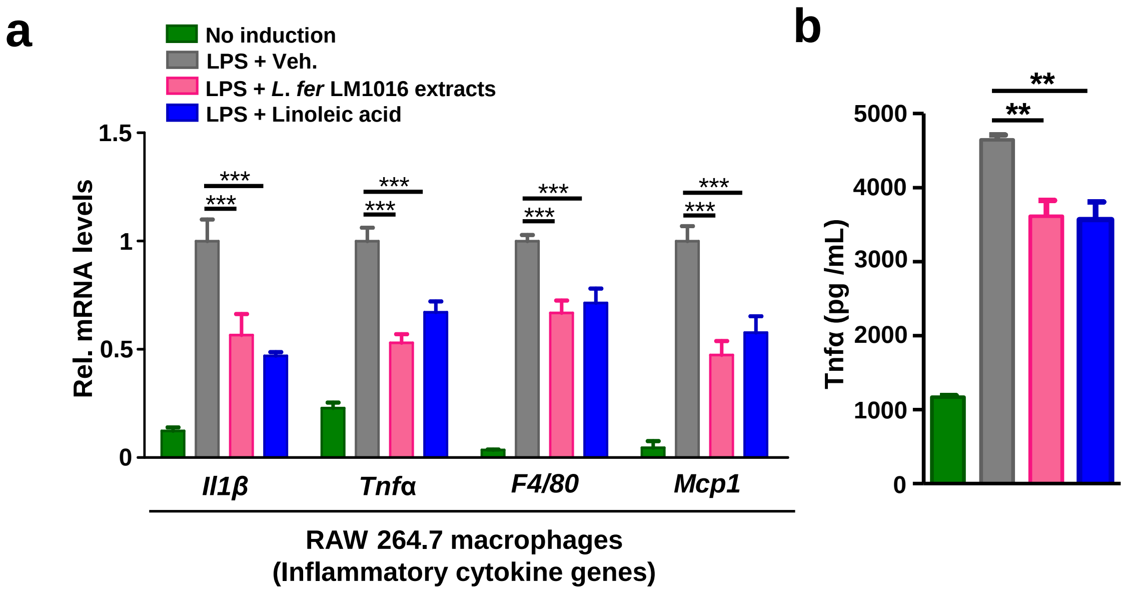

Supplementary Figure S4. Treatment of both *L. fermentum* LM1016 bacterial extracts and linoleic acid dramatically reduces inflammatory signaling in RAW 264.7 macrophage cells. a, b, RAW264.7 macrophage cells were treated with LPS, L. fermentum LM1016 bacterial extracts and linoleic acid for 24 h. (**a**) Gene expression profiles of inflammatory cytokines, including IL-1β, TNFα, F4/80, and Mcp1. (**b**) Protein level of TNFα in the culture medium of RAW264.7 macrophage cells. **$p < 0.01$, ***$p < 0.001$, as determined by one-way ANOVA. Data are expressed as means ± SEMs.

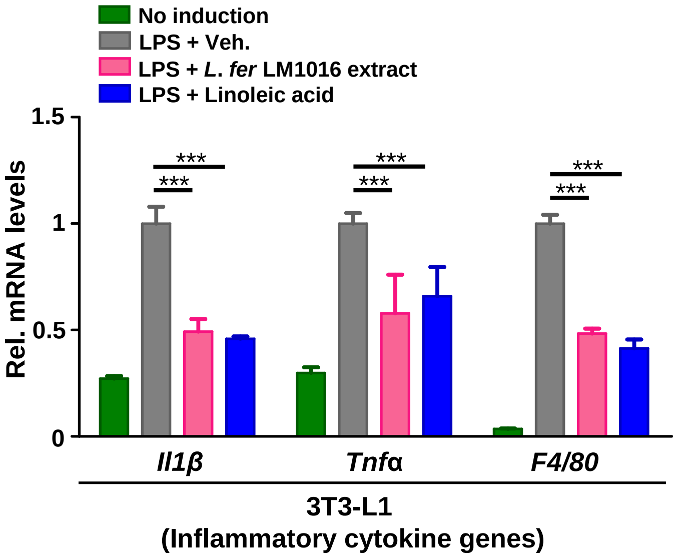

Supplementary Figure S5. Treatment of both *L. fermentum* LM1016 bacterial extracts and linoleic acid dramatically reduces expression of genes involved in inflammatory cytokines in 3T3-L1 cells. 3T3-L1 cells were treated with LPS, *L. fermentum* LM1016 bacterial extracts, and linoleic acid for 24 h. Expression of genes, including IL-1β, TNFα, and F4/80 was largely downregulated by treatment of both *L. fermentum* LM1016 bacterial extracts and linoleic acid. ***$p < 0.001$, as determined by one-way ANOVA. Data are expressed as means ± SEMs.

Supplementary Table 1. Primer list for qPCR

Bacteria	Forward	Reverse	Probe
L. fermentum	5′-GCACCTGATTGATTTTGGTCG-3′	5´-GGTATTAGCATCTGTTTCCAAATG-3´	5´-FAM-CCAACGAGTGGCGGACGGGTGAG-BHQ1-3´
16S rRNA	5´-AGAGTTTGATCCTGGCTCAG-3´	5´-CTGCTGCCTYCCGTA-3´	5´-FAM-TAACACATGCAAGTCGA-BHQ1-3´

Gene	Forward	Reverse
Mcp1	5′-GGCTCAGCCAGATGCAGTTAAC-3′	5′-AGCCTACTCATTGGGATCATCTTG-3′
Ifng	5′-ATGAACGCTACACACTGCATC-3′	5′-CCATCCTTTTGCCAGTTCCTC-3′
Il-1b	5′-CGGCACACCCACCCTG-3′	5′-AAACCGCTTTTCCATCTTCTTCT-3′
Il-18	5′-CAGGCCTGACATCTTCTGCAA-3	5′-TCTGACATGGCAGCCATTGT-3′
Tgfb	5′-CTTCAATACGTCAGACATTCGGG-3′	5′-GTAACGCCAGGAATTGTTGCTA-3′
F4/80	5′-TGACAACCAGACGGCTTGTG-3′	5′-GCAGGCGAGGAAAAGATAGTGT-3′
Dio2	5′-AATTATGCCTCGGAGAAGACCG-3′	5′-GGCAGTTGCCTAGTGAAAGGT-3′
Acadm	5′-AGGGTTTAGTTTTGAGTTGACGG-3′	5′-CCCCGCTTTTGTCATATTCCG-3′
Esrra	5′-AGGTGGACCCTTTGCCTTTC-3′	5′-GGCATGGCGTACAGCTTCT-3′
Ucp1	5′-AGGCTTCCAGTACCATTAGGT-3′	5′-CTGAGTGAGGCAAAGCTGATTT-3′
Aox	5′-TAACTTCCTCACTCGAAGCCA-3′	5′-AGTTCCATGACCCATCTCTGTC-3′
Cyp7a1	5′-TCATTGCTTCAGGGCTCCTG-3′	5′-TGGGCATCTCAAGCAAACAC-3′
Cyp27a1	5′-CCAGGCACAGGAGAGTACG-3′	5′-GGGCAAGTGCAGCACATAG-3′
GcK	5′-CTGGATGACAGAGCCAGGATG-3′	5′-AGTTGGTTCCTCCCAGGTCT-3′
Pepck	5′-AAAAGCCTTTGGTCAACAAC-3′	5′-AAACTTCATCCAGGCAATGT-3′
Pparg	5′-TCGCTGATGCACTGCCTATG-3′	5′-GAGAGGTCCACAGAGCTGATT-3'
Srebp1c	5′-GAAGCTGTCGGGGTAGCGTCT-3′	5′-CTCTCAGGAGAGTTGGCACCTG-3′
Acc	5′-GGACAGACTGATCGCAGAGAAAG-3′	5′-TGGAGAGCCCCACACACA-3′
Fasn	5′-GCTGCGGAAACTTCAGGAAAT-3′	5′-AGAGACGTGTCACTCCTGGACTT-3′

Scd1	5′-TTCTTGCGATACACTCTGGTGC-3′	5′-CGGGATTGAATGTTCTTGTCGT-3′
Acacb	5′-CGCTCACCAACAGTAAGGTGG-3′	5′-GCTTGGCAGGGAGTTCCTC-3′
Dgat2	5′-GCGCTACTTCCGAGACTACTT-3′	5′-GGGCCTTATGCCAGGAAACT-3′
Chrebp	5′-CATTGCCAACATAAGCATCTTC-3′	5′-GTCCGATATCTCCGACACACTC-3′
Cidec	5′-ATGGACTACGCCATGAAGTCT-3′	5′-CGGTGCTAACACGACAGGG-3′
G0S2	5′-TAGTGAAGCTATACGTTCTGGGC-3′	5′-GTCTCAACTAGGCCGAGCA-3′
36B4	5′-CGTCCTCGTTGGAGTGACA-3′	5′-CGGTGCGTCAGGGATTG-3′
Ibabp	5´-CTTCCAGGAGACGTGATTGAAA-3´	5´-CCTCCGAAGTCTGGTGATAGTTG-3´
Fgf-15	5´-ATGGCGAGAAAGTGGAACGG-3´	5´-CTGACACAGACTGGGATTGCT -3´
Ostβ	5´-GTATTTTCGTGCAGAAGATGCG-3´	5´-ATTTCTGTTTGCCAGGATGCTC-3´
G6pase	5´-TTTCGCGCTTGGATTCTACCTGC-3´	5´-GTGGACCCATTCTGGCCGCTC-3´
adiponectin	5´-AGCCTGGAGAAGCCGCTTAT-3´	5´-TTGCAGTAGAACTTGCCAGTGC-3´

Supplementary Table 2. *Lactobacillus* strains used in this study

Species	Strain	Abbreviation
Lactobacillus fermentum	LM1016	*L. fer* LM1016
Lactobacillus acidophilus	Rosell418	*L. aci* Rosell418
Lactobacillus acidophilus	La14	*L. aci* La14
Lactobacillus salvarius	Lrs-33	*L. sal* Lrs33
Lactobacillus rhamnosus	HOWARU Rhamnosus	*L. rha* HOWARU
Lactobacillus paracasei	LPC37	*L. par* LPC37
Lactobacillus helveticus	LH166	*L. hel* LH166
